# Comparative Targeted Metabolomics of Ischemic Stroke: Thrombi and Serum Profiling for the Identification of Stroke-Related Metabolites

**DOI:** 10.3390/biomedicines12081731

**Published:** 2024-08-02

**Authors:** Ivana Karmelić, Ivana Rubić, Katarina Starčević, David Ozretić, Zdravka Poljaković, Mia Jurilj Sajko, Vladimir Kalousek, Rafaela Kalanj, Dina Rešetar Maslov, Josipa Kuleš, Marina Roje Bedeković, Tomislav Sajko, Krešimir Rotim, Vladimir Mrljak, Dragana Fabris

**Affiliations:** 1 Department of Medical Chemistry, Biochemistry and Clinical Chemistry, School of Medicine, University of Zagreb, Šalata 3, 10000 Zagreb, Croatia; 2 Laboratory of Proteomics, Clinic for Internal Diseases, Faculty of Veterinary Medicine, University of Zagreb, Heinzelova 55, 10000 Zagreb, Croatia; 3 Department of Neurology, University Hospital Centre “Zagreb”, Kišpatićeva 12, 10000 Zagreb, Croatia; 4 Department of Diagnostic and Interventional Neuroradiology, University Hospital Centre “Zagreb”, Kišpatićeva 12, 10000 Zagreb, Croatia; 5 Department of Neurosurgery, University Hospital Centre “Sestre Milosrdnice”, Vinogradska cesta 29, 10000 Zagreb, Croatia; 6 Department of Radiology, University Hospital Centre “Sestre Milosrdnice”, Vinogradska cesta 29, 10000 Zagreb, Croatia; 7 Department of Neurology, University Hospital Centre “Sestre Milosrdnice”, Vinogradska cesta 29, 10000 Zagreb, Croatia; 8 Department of Chemistry and Biochemistry, Faculty of Veterinary Medicine, University of Zagreb, Heinzelova 55, 10000 Zagreb, Croatia

**Keywords:** ischemic stroke, stroke thrombi, targeted metabolomics, metabolic biomarkers

## Abstract

Ischemic stroke is one of the leading causes of death and permanent disability in the world. Rapid diagnosis and intervention are crucial for reducing its consequences on individuals and societies. Therefore, identifying reliable biomarkers for early detection, prognostics, and therapy can facilitate the early prediction and prevention of stroke. Metabolomics has been shown as a promising tool for biomarker discovery since many post-ischemic metabolites can be found in the plasma or serum of the patient. In this research, we performed a comparative targeted metabolomic analysis of stroke thrombi, stroke patient serums, and healthy control serums in order to determine the alteration in the patients’ metabolomes, which might serve as biomarkers for early prediction or stroke prevention. The most statistically altered metabolites characterized in the patient serums compared with the control serums were glutamate and serotonin, followed by phospholipids and triacylglycerols. In stroke thrombi compared with the patients’ serums, the most significantly altered metabolites were classified as lipids, with choline-containing phospholipids and sphingomyelins having the highest discriminatory score. The results of this preliminary study could help in understanding the roles of different metabolic changes that occur during thrombosis and cerebral ischemia and possibly suggest new metabolic biomarkers for ischemic stroke.

## 1. Introduction

Stroke remains one of the leading causes of death and permanent disability in the world, with ischemic stroke (IS) representing over 80–85% of all incident strokes [1]. The stroke frequency is expected to grow with the increasing age of the population [2]. Rapid diagnosis and intervention are essential to reduce mortality and morbidity [3].

Mechanical thrombectomy (MT) is an endovascular procedure involving the recanalization of an intracranial occlusion by removing a thrombus using a retrievable stent or aspiration catheter [4]. Until the development of MT in 2015, intravenous thrombolysis was the only medical therapy shown to improve patient outcomes in acute ischemic stroke [5], but with a narrow treatment window and numerous complications ranging from brain hemorrhage, cardiac complications, seizures, and deep vein thrombosis in later stages of treatment [6]. The composition and structural organization of stroke thrombi were not researched until recent years because of the unavailability of the thrombus material. The development of MT has enabled the retrieval of stroke thrombi and allowed the possibility of detailed analysis of thrombi composition and structure, which is crucial for improving pharmacological or endovascular recanalization efficiency in acute stroke treatment [7].

Metabolomics is a key tool for analyzing end products of cellular metabolism that serve as dynamic and sensitive indicators of physiological distress, reflecting the interaction among genetic predispositions, environmental factors, and disease states [8]. Because of the impaired integrity of the blood–brain barrier in the ischemic brain, many of the post-ischemic metabolites are more easily found in the plasma or serum of the patient [9]. Recently, increased research interest in the metabolomics of cerebrovascular diseases and stroke has identified altered serum metabolic profiles in patients having these complex pathologies [10,11,12,13,14,15]. Although the specific IS biomarker has not been identified yet, these studies have pointed out several important stroke-related pathophysiological mechanisms such as excitotoxicity, oxidative stress, and inflammation, all producing metabolites that are to some extent related to cerebral ischemia [16]. Metabolomics research on stroke thrombi in IS has been performed in several studies so far [10,17], indicating that several metabolic pathways are altered in IS. Nevertheless, the data on stroke thrombi metabolomics is still very limited. In this preliminary research, we performed a detailed, targeted metabolomics analysis of stroke thrombi, corresponding stroke patient serums, and healthy control serums, with special interest in lipid metabolites because of the well-known influence of lipids in the pathology of atherosclerosis and cerebrovascular diseases. This study aimed to use comparative metabolomics of stroke thrombi, as insufficiently analyzed biomaterial, stroke patient serums, and healthy controls to differentiate the most significant metabolites that might serve as metabolic biomarkers of ischemic stroke. The results of this study could contribute to a better understanding of metabolic alterations underlying cerebral ischemia, which could help in developing new therapeutic strategies for ischemic stroke.

## 2. Materials and Methods

### 2.1. Study Subjects

Patients enrolled in this study were urgently admitted to University Hospital Centre “Zagreb” Neurology Department and University Hospital Centre “Sestre milosrdnice” Radiology Department in Zagreb, Croatia, receiving mechanical thrombectomy (MT) with or without bridging intravenous thrombolytic therapy (IVT). They were selected based on our University Centre stroke guidelines, which are mainly based on the European Stroke Organization (ESO) mechanical thrombectomy guidelines published in 2019 [18], the ESO thrombolysis guidelines published in 2021 [19], and the ESO thrombolysis recommendation before MT published in 2022 [20].

All included patients (N = 14) were adults (>18 years), had a focal neurologic deficit defined as at least 4 points on the National Institute of Health Stroke Scale (NIHSS), and a duration of symptoms up to 24 h. Non-contrast computed tomography (CT) was used to exclude cerebral hemorrhage or other causative intracranial pathology other than ischemia. CT angiography confirmed intracranial large vessel occlusion of the anterior or posterior circulation (internal carotid artery [ICA], carotid T, M1, and M2 segments of the middle cerebral artery [MCA], A1 segment of the anterior cerebral artery [ACA], P1 segment of the posterior cerebral artery [PCA], basilar artery [BA], and vertebral artery [VA]) and an Alberta Stroke Program Early CT Score (ASPECT) between 6 and 10. Patients that arrived later than 6 h from symptom onset and underwent MT had CT perfusion imaging demonstrating core/penumbra mismatch defined as core volume less than 70 mL, critically hypoperfused volume/core ratio larger than 1, 2, and mismatch volume larger than 10 mL. Patients who received bridging IVT had symptom duration of up to 4–5 h or perfusion mismatch on CT perfusion imaging according to the aforementioned criteria. All patients who received IVT were administered with alteplase (0.9 mg/kg). The exclusion criteria for the patients enrolled in this study was a therapy that influences serum lipid profile or contraception therapy. Mechanical thrombectomy was performed using all three of the following main techniques: stent retriever technique, contact aspiration, and combined technique.

Participants in the control group (N = 14) were selected from the Department of Neurosurgery, University Hospital Centre “Sestre milosrdnice”, Zagreb, Croatia. Baseline assessments were conducted for all participants, including demographic information, medical history, and relevant clinical measures, ensuring comparability with the stroke group as closely as possible. In the control group, exclusion criteria included a history of stroke and any other cerebrovascular events or neurological disorders (other than stroke), severe cardiovascular diseases, confirmed chronic inflammatory conditions, confirmed severe renal or hepatic diseases, and uncontrolled hypertension as well as pregnancy and the intake of medications that could interfere with the study outcomes.

This study was conducted under the Declaration of Helsinki and approved by the Ethics Committee of the University Hospital Centre “Zagreb” (protocol code 02/21 AG, 28 October 2019) and University Hospital Centre “Sestre milosrdnice” (protocol code EP-18031/19-12, 23 January 2020) and by the Ethics Committee of the School of Medicine, University of Zagreb (protocol code 380-59-10106-20-111/15 and 23 January 2020). All study subjects signed an informed consent.

### 2.2. Clinical and Biochemical Measurements

For clinical laboratory and targeted metabolomics analysis, venous blood samples were collected after 12 h fasting for the control group and immediately before IVT and MT for the patient group. The serum aliquots were stored in glass tubes at −80 °C until measurements were performed.

Biochemical parameters were measured at the Biochemistry Laboratory of University Hospital Centre “Zagreb” and University Hospital Centre “Sestre milosrdnice” in Zagreb, Croatia. Biochemical analyses of serum samples in the clinical setting involved measurement of blood glucose and the lipid profile (total cholesterol [tCH], triglycerides [TGs], HDL-cholesterol [HDL-CH], LDL-cholesterol [LDL-CH]) as well as the coagulation profile (prothrombin time [PT], activated partial thromboplastin time [aPTT]), international normalized ratio [INR]) by standard laboratory procedures [21,22]. Body mass index (BMI) was calculated by the standard formula BMI = weight (kg)/height (m^2^). Systolic and diastolic blood pressure were measured using a sphygmomanometer.

### 2.3. Preparation of Stroke Thrombi for Targeted Metabolomics Analysis

After the MT procedure, each thrombus sample was washed with a cold saline solution and stored in glass tubes at −80 °C until metabolomics analysis. Collected stroke thrombi were weighted on ice, and a 3-fold volume of ice-cold isopropanol was used as the extraction solvent (Table S1). The samples were vortexed and sonicated on ice 3 times for 30 s at max amplitude, spin-down between cycles, and held on ice for 1 min. After the third sonication cycle, samples were vortexed for 30 s and centrifuged at 10,000× *g* for 5 min at 4 °C. The supernatants were used for further analysis on the AbsoluteIDQ p400 kit (Biocrates Life Science AG, Innsbruck, Austria) with mass spectrometry (Q Exactive Plus hybrid quadrupole—Orbitrap; Thermo Fisher Scientific, Bremen, Germany).

### 2.4. Targeted Metabolomics Analysis

The concentration of 408 endogenous metabolites divided into 11 analytical classes, including amino acids (N = 21), biogenic amines (N = 21), and monosaccharides comprising the sum of hexoses, including glucose (N = 1), phosphatidylcholines (N = 172), lysophosphatidylcholines (N = 24), sphingomyelins (N = 31), ceramides (N = 9), diglycerides (N = 18), triglycerides (N = 42), cholesteryl esters (N = 14), and acylcarnitines (N = 55) were measured in stroke patient serums, control serums, and stroke thrombi samples with the Biocrates AbsoluteIDQ p400 kit using mass spectrometry (MS) according to the manufacturer’s protocol.

Sample preparation and metabolite extraction were performed according to the manufacturer instructions provided with the kit and as described in detail previously [23,24,25].

Mass spectrometry analysis was performed on a Dionex Ultimate 3000 UHPLC system (Thermo Fisher Scientific, Germering, Germany) coupled to a Q Exactive Plus hybrid quadrupole–Orbitrap mass spectrometer using an electrospray ionization source. The Thermo p400 HR UHPLC column provided with the kit was used to separate metabolites from the samples.

The MetIDQ 5.2.2. software package Version Boron, an integral part of the Absolute IDQ p400 kit, was used for data processing, quality assessment, and data export. 

Metabolites (amino acids and biogenic amines) were quantified using liquid chromatography–mass spectrometry (LC-MS/MS) via XCalibur Quan 4.1 software (Thermo Fisher Scientific, Waltham, MA, USA) based on a seven-point calibration curve and isotope-labeled internal standards for most analytes, while metabolites (acylcarnitines, glycerophospholipids, glycerides, hexoses, cholesteryl esters, and sphingolipids) were quantified using flow-injection analysis–mass spectrometry (FIA-MS/MS) analysis based on a single-point calibrator with the representative internal standards. All reagents used in this analysis were of LC-MS grade and purchased from Merck (Darmstadt, Germany), Honeywell (Charlotte, NC, USA), and Sigma-Aldrich (St. Louis, MO, USA). Blank samples were used to calculate the limits of detection (LOD). In terms of quantification, if the compounds were quantified with restriction, then the calibration curves had expected coefficients of determination (R^2^) < 0.99 according to the manufacturer’s guidelines. When specific standards were not commercially available and verification of the accuracy was not possible by the manufacturer, the measuring was performed “semi-quantitatively”.

### 2.5. Statistical Analyses

MedCalc 22.019 (Frank Schoonjans, Mariakerke, Belgium) was used for descriptive statistical analysis and for the comparison of clinical and biochemical data between the study groups using Student’s *t*-test or the Mann–Whitney test, depending on distribution normality. 

MetaboAnalyst v.4.0 software (http://www.metaboanalyst.ca, accessed on 15 November 2023) was used for univariate and multivariate statistical analysis of metabolites. Metabolites with 50% missing values were removed and replaced with LoDs (1/5 of the minimum positive value of each variable). The concentrations of each metabolite were normalized by the median, log-transformed, and Pareto-scaled. The overall differences in the metabolomics profile between control and stroke patient serum samples and between stroke patient serums and stroke thrombi were analyzed by Student’s *t*-test, a partial least squares discriminant analysis (PLS-DA), variable importance on projection (VIP), and hierarchical clustering analysis (HCA). A *p*-value ≤ 0.05 was considered statistically significant for all statistical analyses. 

## 3. Results

### 3.1. Clinical Features of the Study Population

The clinical and laboratory parameters of study participants are shown in Table S2 and Table 1. There were no differences in DBP, SBP, or BMI between the groups, but ischemic stroke (IS) patients were significantly older than controls (71.43 ± 12.26 vs. 51.29 ± 15.09). Clinical laboratory values of lipid parameters (TG, tCH, LDL-CH, and HDL-CH) did not show significant differences between groups, whereas glucose showed significant alterations between the patient and control groups. The comparison of coagulation factors (PV, aPTV, and INR) between the IS and control groups revealed that there were no significant differences between groups.

### 3.2. Clinical Features of the Study Population

#### 3.2.1. Comparison of Stroke Patient Serums vs. Control Serums

To determine differences in the serum metabolome, metabolite concentrations in ischemic stroke (IS) patient serums were compared with healthy control serums. The targeted approach (Biocrates analysis) detected 193 metabolites (above the limit of detection, LOD) between the two groups (Table S3), which were classified into groups of amino acids (N = 21), biogenic amines (N = 9), hexoses, including glucose (N = 1), acylcarnitines (N = 4), cholesteryl esters (N = 6), diglycerides (N = 9), triglycerides (N = 29), lysophosphatidylcholines (N = 9), phosphatidylcholines (N = 50), ether phosphatidylcholines (N = 27), sphingomyelins (N = 26) and ceramides (N = 2). Using MetIDQ™ software, 159 lipid metabolites were relatively quantified and 34 metabolites were absolutely quantified. The list of 193 metabolites was used for further statistical analysis.

**Figure 1:**
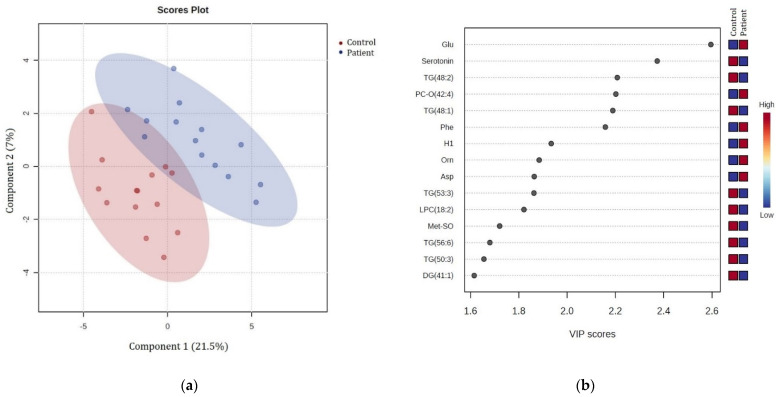
(**a**) Partial least squares discriminant analysis (PLS-DA) score plots were performed on the patient group (blue circle; N = 14) and control group (red circle; N = 14) serums using a targeted metabolomics approach. (**b**) Variable importance in projection (VIP) score plot showing the relative abundance of the 15 most significant metabolites identified by PLS-DA that differ between the patient and control serums. The colored boxes on the right indicate the relative abundance of a metabolite; red boxes indicate high abundance and blue boxes indicate low abundance of a specific metabolite. Glu—glutamate; TG—triglyceride; PC-O—ether phosphatidylcholine; Phe—phenylalanine; H1—hexoses (including glucose); Orn—ornithine; Asp—aspartate; LPC—lysophosphatidylcholine; Met-SO—methionine sulfoxide; DG—diglyceride.

The multivariate partial least squares discriminant analysis (PLS-DA) was performed to identify the metabolites that were the most discriminant between the patient group (N = 14) and control group (N = 14) serums (Figure 1a). This analysis showed a relatively clear intergroup separation of the patient group from the control group but showed overlapping of the two analyzed groups at the same time. The validity of PLS-DA was confirmed by cross-validation and indicated that the best classifier model comprised three components (R^2^ = 0.90, Q^2^ = 0.29).

The overall variable importance in the projection (VIP) score plot generated from the PLS-DA model selected and ranked 15 individual metabolites that contributed significantly to the discrimination between the patient and control groups (Figure 1b). Higher VIP scores suggest greater importance for discrimination between groups. Among them, glutamate, serotonin, triglycerides (TG [48:2] and TG [48:1]), ether phosphatidylcholine (PC-O [42:4]), and phenylalanine mostly contributed to the metabolic differences between the two analyzed groups (VIP > 2), with glutamate, ether phosphatidylcholine (PC-O [42:4]), and phenylalanine having higher abundance and serotonin and triglycerides (TG [48:2] and TG [48:1]) having lower abundance in stroke patient serums compared with the control samples (Figure 1b).

The univariate analysis (Student’s *t*-test) resulted in the identification of 37 metabolites with significantly different concentrations between the patient and control serums (Table S4).

**Figure 2:**
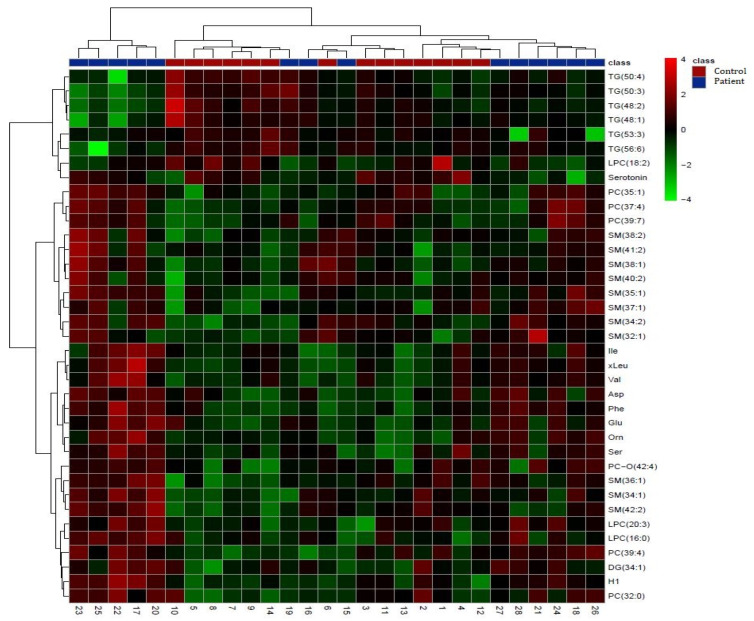
Hierarchical cluster analysis (HCA) of the significantly different metabolites obtained using a *t*-test (*p* < 0.05) between the two groups (stroke patient serums—green panel; control serums—red panel). Each colored cell on the map corresponds to the intensity value, red color—max metabolite concentration, green color—min metabolite concentration. The data were normalized by the median, log-transformed, and Pareto-scaled. TG—triglyceride, PC—phosphatidylcholine; SM—sphingomyelin; Ile—isoleucine, xLeu—leucine; Val—valine; Asp—aspartate; Phe—phenylalanine; Glu—glutamate; Orn—ornithine; Ser—serine, PC-O—ether phosphatidylcholine; LPC—lysophosphatidylcholine; DG—diglyceride; H1—hexoses (including glucose).

Hierarchical cluster analysis (HCA) was performed using 37 significant metabolites from the Student’s *t*-test to obtain visualization of the differentially abundant metabolites and the alteration in serum metabolites between the two groups in the form of a heat map (Figure 2). From the 37 significantly altered metabolites, all amino acids, sphingomyelins, phosphatidylcholines, ether phosphatidylcholines, two lysophosphatidylcholines (LPC [16:0] and LPC [20:3]), and diglyceride, as well as hexoses (including glucose), were significantly increased in the stroke patient serums compared with the control serums. On the other hand, lysophosphatidylcholine (LPC [18:2]), serotonin, and all triglycerides were significantly decreased in the stroke patient serums compared with the control serums (Figure 2). The results that referred to two main clusters were related to the compared samples and metabolites using Euclidean as a distance measure and ward as a clustering algorithm. Sample group clustering for the targeted metabolomics with overlapping of some stroke patient samples and control samples are shown in Figure 2. Interestingly, the metabolomics pattern of the control group (samples 3, 11, 13, 2, 1, 4, and 12) was closer to the group of patients. On the other hand, the metabolomics pattern of samples 23, 25, 22, 17, 20, 19, and 16 in the patient group was closer to the control group.

**Figure 3:**
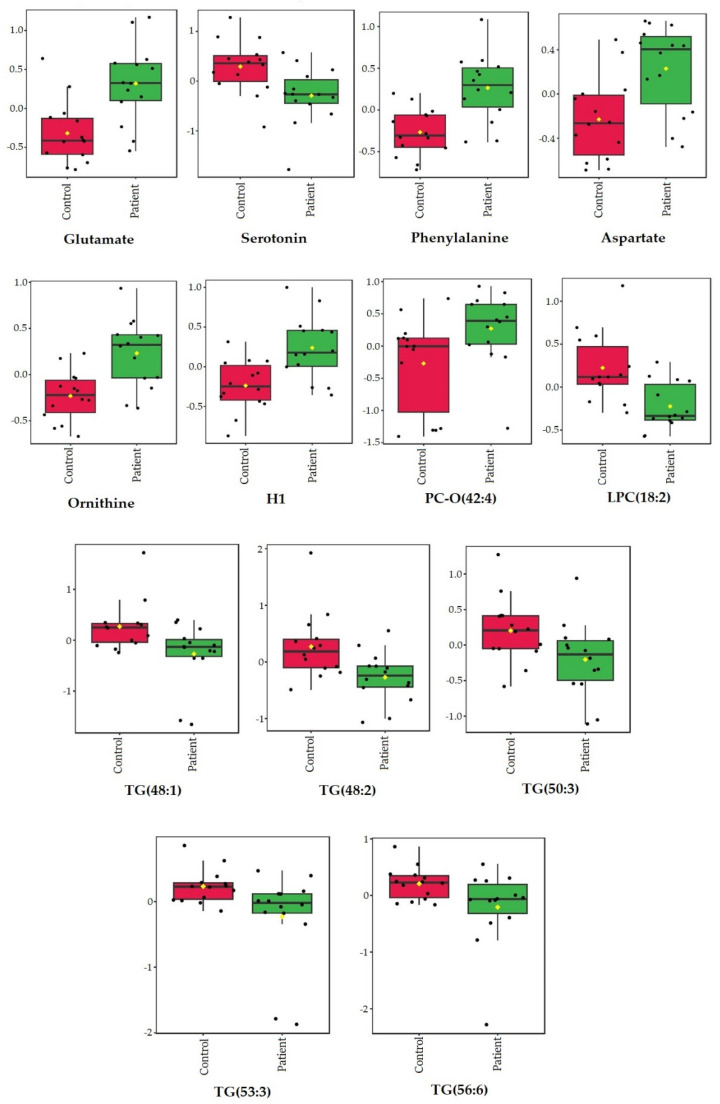
Concentrations of significantly altered metabolites in stroke patient serums vs. control serums that overlap in univariate (Student’s *t*-test) and multivariate (PLS-DA) analyses. Data are presented as box and whisker plots (mean ± SD). Red—control serums; green—stroke patient serums; TG—triglyceride; PC-O—ether phosphatidylcholine; LPC—lysophosphatidylcholines; H1—hexoses (including glucose).

According to both the multivariate and univariate analyses, the concentrations of the following metabolites were statistically altered between the patient and control serums: glutamate, phenylalanine, ornithine, aspartate, serotonin, hexose, triglycerides (TG [48:1], TG [48:2], TG [50:3], TG [53:3], and TG [56:6]), lysophosphatidylcholines (LPC [18:2]), and ether phosphatidylcholine (PC-O [42:4]) (Figure 3).

#### 3.2.2. Comparison of Patient Stroke Thrombi vs. Stroke Patient Serum

The stroke thrombi metabolome from IS patients was compared with the serum metabolome of the same patient. The targeted analysis detected 125 metabolites (above the limit of detection, LOD) between the two groups, which were used for further statistical analysis and classified into groups of amino acids (N = 21), biogenic amines (N = 4), hexoses, including glucose (N = 1), acylcarnitines (N = 2), cholesteryl esters (N = 1), diglycerides (N = 4), triglycerides (N = 8), lysophosphatidylcholines (N = 3), phosphatidylcholines (N = 34), ether phosphatidylcholines (N = 22), sphingomyelins (N = 23), and ceramides (N = 2) (Table S4). Among them, 97 metabolites were relatively quantified, 27 metabolites were absolutely quantified, and 1 metabolite was quantified with restrictions.

**Figure 4:**
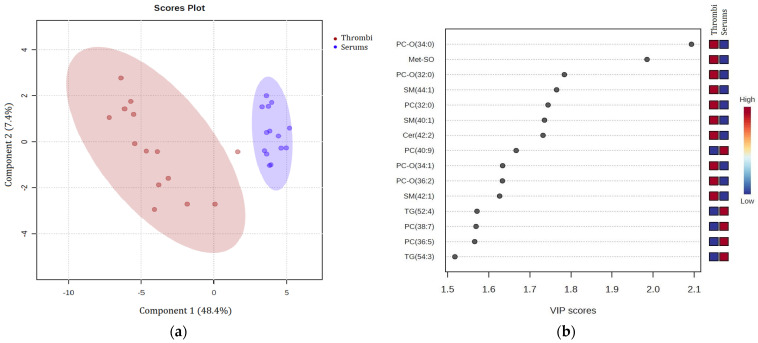
(**a**) Partial least squares discriminant analysis (PLS-DA) score plots performed in stroke thrombi samples (N = 14; red circle) and serum samples (N = 14; blue circle) using a targeted metabolomics approach; (**b**) Variable importance in projection (VIP) score plot showing the relative abundance of the 15 most significant metabolites identified by PLS-DA that differ between stroke thrombi and serum samples. The colored boxes on the right indicate the relative abundance of a metabolite; red boxes indicate high abundance and blue boxes indicate low abundance of a specific metabolite. PC-O—ether phosphatidylcholine; Met-SO—methionine sulfoxide; SM—sphingomyelin; PC—phosphatidylcholine; Cer—ceramide; TG—triglyceride.

To identify metabolites that were the most discriminant between the stroke thrombi group (N = 14) and stroke patient serum group (N = 14), PLS discriminant analysis was performed. The validity of PLS-DA was confirmed by cross-validation and showed that the best classifier model comprised two components (R^2^ = 0.89, Q^2^ = 0.84). This analysis showed a clear intergroup separation of the two groups (Figure 4a). The list of the 15 most important metabolites that contributed significantly to the discrimination between two groups determined by overall VIP score is presented in Figure 4b. Among them, ether phosphatidylcholine (PC-O [34:0]) was the most influential metabolite with the VIP > 2 having a higher abundance in the stroke thrombi samples than in the serum samples.

**Figure 5:**
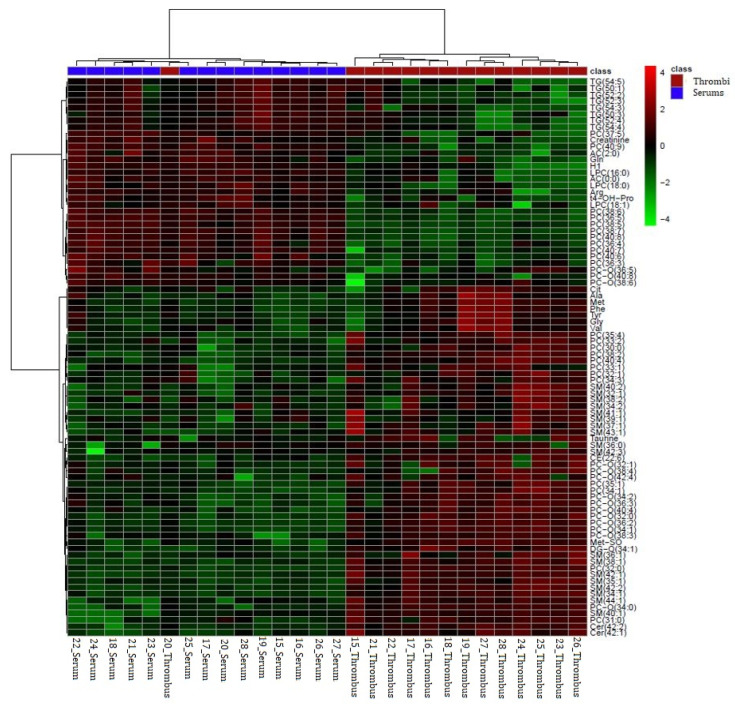
Hierarchical cluster analysis (HCA) of the significantly different metabolites obtained using a *t*-test (*p* < 0.05) between two groups (stroke thrombi—red panel; stroke patient serum samples—green panel). Each colored cell on the map corresponds to the intensity value, red color—max metabolite concentration, green color—min metabolite concentration. The data were normalized by the median, log-transformed, and Pareto-scaled. TG—triglyceride, PC—phosphatidylcholine; PC-O—ether phosphatidylcholine; Gln—glutamine, Arg—arginine; LPC—lysophosphatidylcholine; SM—sphingomyelin; Cer—ceramide; DG—diglyceride; AC—acylcarnitine; t4-OH-Pro—*trans*-4-hydroxy-L-proline; Cit—citruline; Ala—alanine; Met—methionine; Phe—phenylalanine; Tyr—tyrosine; Gly—glycine; Val—valine; Met-SO—methionine sulfoxide Met-SO—methionine sulfoxide; H1—hexoses (including glucose).

Of the 125 detected metabolites, 68.8% (N = 86) had significantly different concentrations between the stroke thrombi samples and serum samples of the same patient according to the univariate analysis (Student’s *t*-test) (Table S6). Hierarchical cluster analysis (HCA) was performed using 86 significant metabolites from the Student’s *t*-test (*p* < 0.05) to represent the differentially abundant metabolites visually between the two groups in the form of a heat map (Figure 5). Among them, all sphingolipids, 23 out of 37 phosphatidylcholines, seven out of nine amino acids, two out of four biogenic amines, cholesteryl esters, and diglycerides were significantly higher in the stroke thrombi compared with the serum samples. On the other hand, all acylcarnitines, triglycerides, lysophosphatidylcholines, and 14 out of 37 phosphatidylcholines were significantly lower in the stroke thrombi compared with the serum samples (Table S5). The results that referred to two main clusters were related to the compared samples and metabolites using Euclidean as a distance measure and ward as a clustering algorithm to perform the cluster analysis. Sample group clustering for the targeted metabolomics with the overlapping of one stroke thrombus sample (sample 20) is shown in Figure 5.

**Figure 6:**
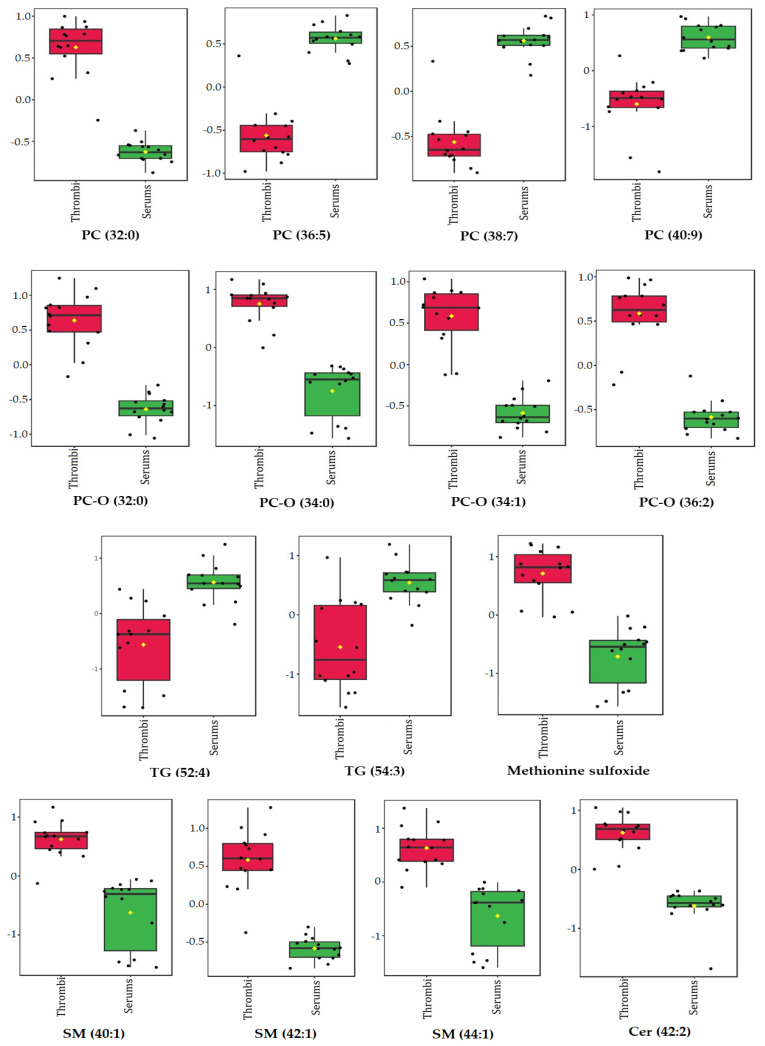
Concentrations of significantly altered metabolites in stroke thrombi samples vs. stroke patient serum samples that overlap in univariate (*t*-test) and multivariate (PLS-DA) analyses. Data are presented as box-and-whisker plots (mean ± SD). Red—stroke thrombi samples, green—stroke patient serum samples. TG—triglyceride; PC-O—ether phosphatidylcholine; LPC—lysophosphatidylcholine; SM—sphingomyelin; PC—phosphatidylcholine; Cer—ceramide.

According to both the VIP scores and Student’s *t*-test, the concentration of the following metabolites was statistically significant between the stroke thrombi and serum samples from the same patients: methionine sulfoxide, triglycerides (TG [52:4] and TG [54:3]), phosphatidylcholines (PC [32:0], PC [36:5], PC [38:7], and PC [40:9]), ether phosphatidylcholines (PC-O [32:0], PC-O [34:0], PC-O [34:1], and PC-O [36:2]), sphingomyelins (SM [40:1], SM [42:1], and SM [44:1]), and ceramide (Cer [42:2]) (Figure 6). 

## 4. Discussion

Because of the impaired integrity of the blood–brain barrier after cerebral ischemia, many of the post-ischemic metabolites are more easily found in the plasma or serum of the patient [9]. Several metabolomic studies performed on stroke thrombi in IS indicated that metabolic pathways such as glycolysis, lactose, and sphingolipid metabolism are altered in IS thrombi [17,18]. In this preliminary study, we performed a comparative targeted metabolomic analysis of 14 stroke thrombi, corresponding stroke patient serums, and healthy control serums and identified a specific subset of stroke-related metabolites. By comparing patient and healthy control serum metabolites using only univariate analysis visualized in the form of a heat map, overlap in the metabolites of the two analyzed groups was shown, which suggests overlapping of some metabolic pathways between the two groups. Therefore, the combination of both univariate and multivariate analyses was performed to identify the metabolites that are discriminatory between the statistically significant groups. We assumed that by comparing stroke thrombi with the stroke patient serums, besides the patient and healthy control serum metabolic comparison, additional information on metabolomics profiles could be found and associated with IS predisposition or diagnostics.

According to both the multivariate and univariate analyses, the concentrations of some metabolites were statistically significant between the patient and control serums, while some were statistically significant between the stroke patient serums and the stroke thrombi.

**Glutamate** concentration was significantly increased in the stroke patient serums compared with the control serums (Figure 2 and Figure 3), with the highest VIP score (VIP > 2.5, Figure 1b), which is in accordance with reported studies. Glutamate is an excitatory amino acid and neurotransmitter that has an important role in mediating neuronal damage during cerebral ischemia [26]. It is considered the main contributor to ischemic brain tissue excitotoxicity as a result of energy failure [16], and higher concentrations of glutamate in the blood and cerebrospinal fluid have been reported and associated with poor clinical outcomes and neurological impairment after stroke [14,27]. 

Contrary to glutamate, the **serotonin** concentration was significantly decreased in the stroke patient serums compared with the control serums (Figure 2 and Figure 3), also having a high VIP score (VIP > 2, Figure 1b). This is in accordance with some early reports that indicated decreased plasma serotonin levels in patients with acute ischemic stroke [28], while other studies reported elevated post-stroke plasma serotonin levels [29] and some reported no significant differences in serotonin levels [30]. Serotonin is a well-known excitatory glutamatergic neurotransmitter that modulates neural activity and regulates mood and behavior, but it is also a very important regulator of platelet aggregation and cardiovascular function [31]. An abnormal serotoninergic mechanism may lead to a pro-thrombotic state, while down-regulation may increase the risk of bleeding [32]. Many studies have shown that selective serotonin reuptake inhibitors (SSRIs) can improve clinical outcomes from ischemic stroke [33] possibly because of the effect on neuronal cell survival, synaptic plasticity, and neuronal connections [34].

The **phenylalanine** concentration was also increased in the patient vs. control serums in this study (Figure 2 and Figure 3) with VIP > 2 (Figure 1b), which is also in accordance with related studies in which it was associated with ischemic stroke and cardiovascular diseases [35] and also suggested to be a compensatory response to high neurotoxic concentrations of glutamate because of phenylalanine inhibition of excitatory glutamatergic synaptic transmission [16]. Also, phenylalanine/tyrosine levels have been reported to be highly elevated in the acute phase of ischemic stroke, suggesting that their ratio is a potential biomarker of acute IS [36]. 

Excessive release of **aspartate**, another excitatory amino acid significantly elevated in the stroke patient serums (Figure 2 and Figure 3), is believed to be a result of membrane depolarization after cerebral ischemia and related to excitotoxicity and oxidative stress [37]. 

In microglial cells, **ornithine** is a key substrate for polyamine biosynthesis, which is involved in the regulation of inflammatory reactions and cell renewal processes after cerebral ischemia [38], but the main metabolic fate for ornithine in the brain is most probably conversion to glutamate and GABA via ornithine aminotransferase [39]. Therefore, the increased ornithine concentration in the stroke patient serums (Figure 2 and Figure 3) suggests both involvement in neuronal damage and cell renewal after cerebral ischemia. Also, ornitine has been positively related to infarct volume in moderate ischemic stroke patients and suggested as an inclusion biomarker for early-stage stroke detection [26].

**Methionine sulfoxide**, a cell oxidative stress product, was significantly elevated in the stroke thrombi compared with the stroke patient serums (Figure 5 and Figure 6; VIP > 1.5, Figure 4b). It was previously reported to be elevated in stroke patients’ plasma and associated with an increased risk of ischemic stroke [40,41].

Besides several significantly altered amino acids that are involved in the ischemic metabolic serum profile, **glucose** concentrations were also significantly increased in the stroke patient serums, as shown by both standard laboratory blood tests (Table 1) and metabolomics serum analysis (Figure 2 and Figure 3; VIP > 1.5, Figure 1b). Similar results were previously reported in many studies that considered glucose to be a predictor of poor stroke outcomes [42]. Elevated serum glucose, nevertheless, may not be a realistic metabolite in this study since the patients were not fasted as they urgently arrived at the hospital. Hyperglycemia was reported to be present in almost 40% of stroke patients [43], and it is suggested to promote ischemic injury by several proposed mechanisms, including acidosis due to anaerobic metabolism of glucose to lactic acid, superoxide and nitric oxide production, enhanced glucose–sodium exchange, abnormal protein glycosylation, and advanced glycation products [44]. 

The only lipid metabolite that was significantly elevated in the stroke patient serums vs. the controls was **ether phosphatidylcholine (PC-O [42:4])** (Figure 2 and Figure 3; VIP > 2, Figure 1b). Ether phospholipids, including plasmalogens, have different biological functions such as modulation of membrane trafficking, cell signaling, oxidative status, and storage of polyunsaturated fatty acids (PUFAs), and their compositions and their contents are altered in the plasma of patients suffering from different brain disorders [45]. Interestingly, all other ether phospholipids in this study (Figure 5 and Figure 6) were characterized in significantly higher concentrations in the stroke thrombi compared with the stroke patient serums, with **PC-O (34:0)** having the highest VIP score (VIP > 2; Figure 4b), pointing out their potential as a stroke biomarker of oxidative stress and brain injury. On the other hand, “classical” phospholipid species such as **phosphatidylcholine** (VIP > 1.5, Figure 4b) were significantly increased in the stroke patient serum samples compared with the stroke thrombi (Figure 5 and Figure 6); moreover, all had a very high abundance of PUFA content. The only exception was **PC (32:0)**, which was the only phosphatidylcholine species that was significantly decreased in the stroke patient serums compared with the stroke thrombi (Figure 5 and Figure 6). Interestingly, the fatty acid composition is identical to that in PC-O (32:0), which is also much more expressed in the thrombi than in the stroke patient serum samples. This may indicate the important role of these particular PCs and PC-Os in either coagulation or ischemia. The only **lysophosphatidyl choline** species characterized as statistically significant and discriminatory in this study was **LPC (18:2)**, which was lower in the stroke patient serum compared with the healthy control serums (Figure 3; VIP > 1.5, Figure 1b). Choline-containing phospholipids are acetylcholine precursors and they have been proposed as adjuvant therapy in acute stroke because of their anti-inflammatory effects and choline’s involvement in membrane synthesis, which is suggested as beneficial for reducing cell injury in the ischemic brain [46].

Clinical laboratory analysis of all lipid parameters (TG, tCH, LDL-CH, and HDL-CH) did not show significant differences between the patient and control serums (Table 1), despite their common association with high risk of atherosclerosis and subsequently ischemic stroke [47]. In accordance with this, in our metabolomics study, there were no significant differences detected in cholesterol or cholesterol ester species between these two groups. On the other hand, **triacylglycerol** species (VIP > 1.5, Figure 1b) were significantly decreased in the stroke patient serums compared with the control serums (Figure 2 and Figure 3), while TG (52:4) and TG (54:3) (VIP > 1.5, Figure 4b) were decreased in the stroke thrombi compared with the stroke patient serums (Figure 5 and Figure 6). This is in accordance with previous studies that characterize TG as bioenergetic compounds of lipid droplets that are also present in neural cells, which were reported to have low abundance in plasma patients with stroke recurrence after transient ischemic attack, possibly because of low accumulation or formation of cerebral lipid droplets as a product of a defective ischemia-associated stress response [48].

Of all the sphingolipid metabolites characterized in this study, **sphingomyelins** (SM [40:1], [42:1], and [44:1]) and **ceramide** Cer (42:2) were detected as significantly higher in the stroke thrombi than in the stroke patient serums (Figure 5 and Figure 6; VIP > 1.5, Figure 4b). Taking into consideration only univariate analysis, of the 26 detected SM species between the patient and control serums, 11 SM species were significantly increased in the stroke patient serums (Figure 2). In thrombin-stimulated platelets, active sphingomyelinase enzyme hydrolyses sphingomyelin to phosphocholine and ceramide, which are involved in signal transduction events during platelet activation [49]. Long-chain ceramides are involved in apoptotic pathways and inflammation related to cerebral ischemia [50] and their elevated serum levels have been reported as predictors of the risk and severity of ischemic stroke [51]. Sphingomyelins, as major constituents of lipid rafts, are involved in signaling cascades, and their deficiency has been shown to suppress the inflammation induced by cerebral ischemia damage after ischemic stroke in mice [52]. The reason why some particular SM or Cer species are elevated or decreased in different pathologies remains unclear.

### Study Limitations

Our study has several limitations. This research was conducted using a relatively small number of subjects, which may influence the statistical power of this study and affect the generalizability of the results because of the heterogeneity in etiologies, comorbidities, clinical presentations, and demographics of ischemic stroke patients. Age differences between the analyzed groups may also influence the accuracy of the compared metabolic profiles. Unfortunately, it was difficult to age-match the control group with the patients because of stringent inclusion criteria. Age-matching without specific health criteria is challenging, especially in older populations. Therefore, this preliminary research could be validated and confirmed in a larger and more diverse study. Also, combining all etiologies of ischemic stroke (including cardioembolism, large-artery atherosclerosis, and other etiologies) may mask important differences in metabolite profiles and metabolic pathways associated with specific subtypes. This limitation may affect the clinical relevance of our findings, as stroke subtypes have varying prognoses and treatment strategies. Future metabolomics analysis of IS thrombi with larger sample sizes should aim to explore metabolomics differences across ischemic stroke subtypes. Since the patients’ blood samples were not collected after overnight fasting, potential nutritional influence on metabolite profile cannot be excluded. Furthermore, other stroke risk factors such as physical activity, smoking, and alcohol consumption were not evaluated.

## 5. Conclusions

The comparative targeted metabolomics analysis of stroke thrombi, corresponding stroke patient serums, and healthy control serums obtained in this preliminary study identified a specific subset of stroke-related metabolites. This study underlined the importance of some previously known metabolites associated with stroke, such as glutamate, phenylalanine, and ornithine, while also detecting a variety of new stroke-related lipid metabolites. Notably, this study identified specific choline-containing phospholipids, sphingomyelins, and ceramides, suggesting their potential roles in coagulation and ischemic processes. Considering the scarce information on the stroke thrombi metabolomics profile present in the literature, these results provide valuable information about thrombi composition in correlation with stroke patient serum metabolites, reflecting the pathophysiological processes that occur as a consequence of cerebral ischemia. These data could help to elucidate the roles of particular metabolites in the coagulation and pathogenesis of ischemic stroke and suggest new candidates for early diagnostic, prognostic, and therapeutic biomarkers of ischemic stroke, potentially leading to better patient outcomes and reduced morbidity.

## Figures and Tables

**Table 1:** Demographic and laboratory characteristics of the study participants.

	Patient	Control	*p*-Value
Age	70.36 ± 12.82	52.29 ± 15.09	0.002
Sex M/F	8/6	8/6	-
DTPT (min)	202.70 ± 114.19	-	-
HST (days)	16.90 ± 9.46	-	-
BMI (kg/m^2^)	26.34 ± 4.07	23.79 ± 5.35	0.192
DBP (mmHg)	79.71 ± 9.21	83.79 ± 14.46	0.382
SBP (mmHg)	146.43 ± 16.70	135.50 ± 14.31	0.074
TG (mmol/L)	1.51 ± 0.47	1.49 ± 0.73	0.907
tCH (mmol/L)	5.17 ± 0.67	5.11 ± 1.10	0.874
LDL-CH (mmol/L)	3.07 ± 1.16	3.09 ± 1.08	0.962
HDL-CH (mmol/L)	1.02 ± 0.19	1.22 ± 0.37	0.079
Glucose (mmol/L)	8.12 ± 1.26	5.73 ± 0.85	<0.0001
PV	0.91 ± 0.17	0.98 ± 0.14	0.251
aPTT	24.37 ± 1.79	23.79 ± 2.48	0.485
INR	1.07 ± 0.10	1.04 ± 0.06	0.464

M, male; F female; DTPT, door-to-groin puncture time; HST, hospital stay time; BMI, body mass index; DBP, diastolic blood pressure; SBP, systolic blood pressure TG, triglyceride, tCH, total cholesterol; HDL-CH, high-density lipoprotein cholesterol; LDL-CH, low-density lipoprotein cholesterol; PT, prothrombin time; aPTT, activated partial thromboplastin time; INR, international normalized ratio. Data are expressed as mean ± standard deviation (SD). Differences between groups were evaluated by Student’s *t*-test because all parameters followed a normal distribution.

## Data Availability

All data are included in this paper and the Supplementary Materials. The datasets acquired during the current study are available from the corresponding authors upon request.

## Supplementary Materials

The following supporting information can be downloaded at https://www.mdpi.com/article/10.3390/biomedicines12081731/s1, Table S1: Stroke thrombi mass and isopropanol volume; Table S2: Clinical and laboratory parameters obtained by standard laboratory procedures in the control and stroke patient serums; Table S3: Concentrations (μmol dm^−3^) of all valid metabolites obtained by targeted metabolomics (AbsoluteIDQ^®^ p400 HR Kit, Biocrates LifeSciences AG, Innsbruck, Austria) in the control and stroke patient serums; Table S4: Significant metabolites with differential abundance between the two groups (serum of control; serum of patients) obtained by LC-MS/MS and FIA-MS/MS analysis; Table S5: Concentrations (μmol dm^−3^) of all valid metabolites obtained by targeted metabolomics (AbsoluteIDQ^®^ p400 HR Kit, Biocrates LifeSciences AG, Innsbruck, Austria) in the serum and stroke thrombi of patients; Table S6: Significant metabolites with differential abundance between the serums and thrombi of patients obtained by LC-MS/MS and FIA-MS/MS analyses.
